# Osteoid osteoma of the rib: A report of two cases

**DOI:** 10.3892/ol.2015.2895

**Published:** 2015-01-26

**Authors:** ZHIPING DENG, YI DING, LIN HAO, FAJUN YANG, LIHUA GONG, YI DING, XIAOHUI NIU

**Affiliations:** 1 Department of Orthopedic Oncology Surgery, Beijing Jishuitan Hospital, Peking University, Beijing 100035, P.R. China; 2 Department of Pathology, Beijing Jishuitan Hospital, Peking University, Beijing 100035, P.R. China

**Keywords:** osteoid osteoma, rib, tumor

## Abstract

Osteoid osteoma is type of benign bone tumor, characterized by a well-demarcated core with a typical size of <1 cm and by a distinctive surrounding zone of reactive bone formation. The tumor can occur anywhere in the cortex or medulla of the skeleton. However, the lesion usually affects the long bones of the lower extremities. The present study describes two cases of osteoid osteomas located in the rib.

## Introduction

Osteoid osteoma is a type of benign bone tumor, which was first described by Jaffe in 1935 (1). It is a rare condition, which accounts for only 3% of primary bone tumors. Individuals aged between 5 and 24 years old are the most commonly affected (2). The main symptom of the disease is pain that worsens at night, which may be alleviated by aspirin. Current treatment modalities include surgical excision, as well as less invasive techniques (3,4). After treatment, symptoms can be controlled. The most commonly affected sites are the long bones of the lower extremities and the patient outcome is good. The ribs are rarely involved. The present study describes two cases of osteoid osteomas located in the rib. Written informed consent was obtained from both patients.

## Case reports

### Case 1

**Figure 1:**
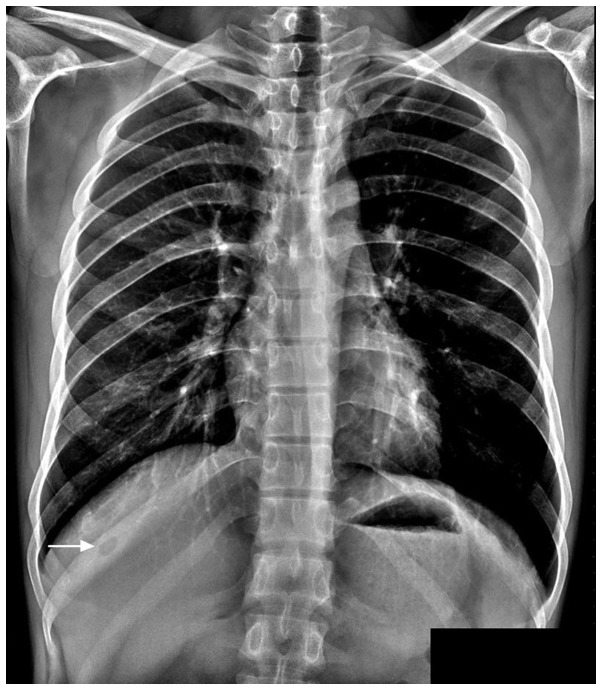
X-ray image at anteroposterior view revealing the presence of a lesion at the tenth right rib.

**Figure 2:**
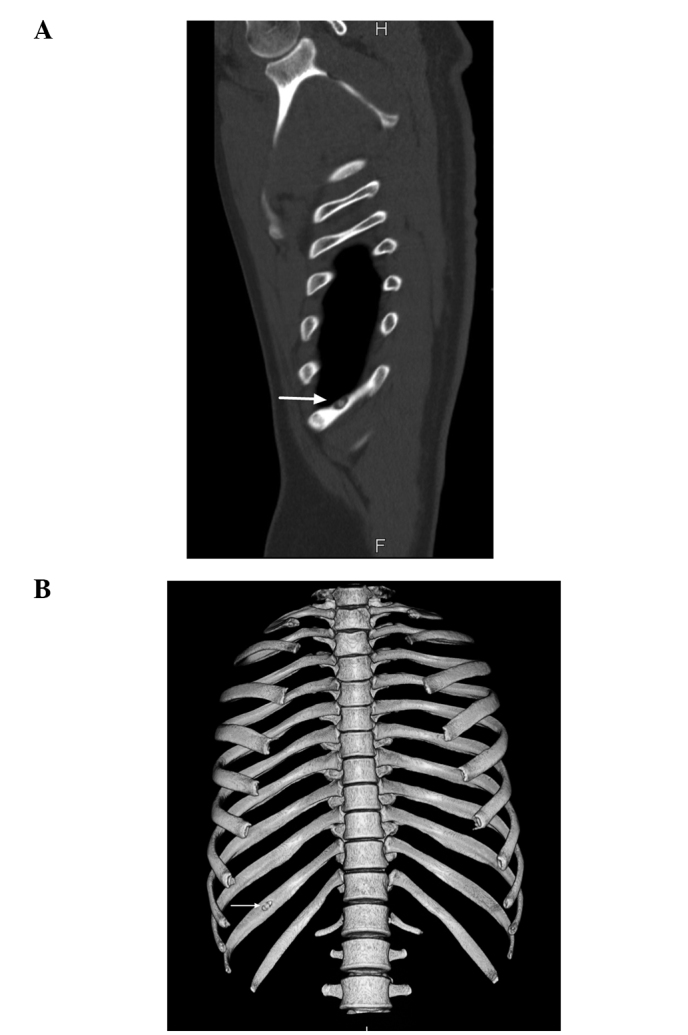
(A) Computed tomography revealing the nidus and central calcification. (B) Three dimensional reconstructed view revealing a lesion located at the visceral side of the rib.

**Figure 3:**
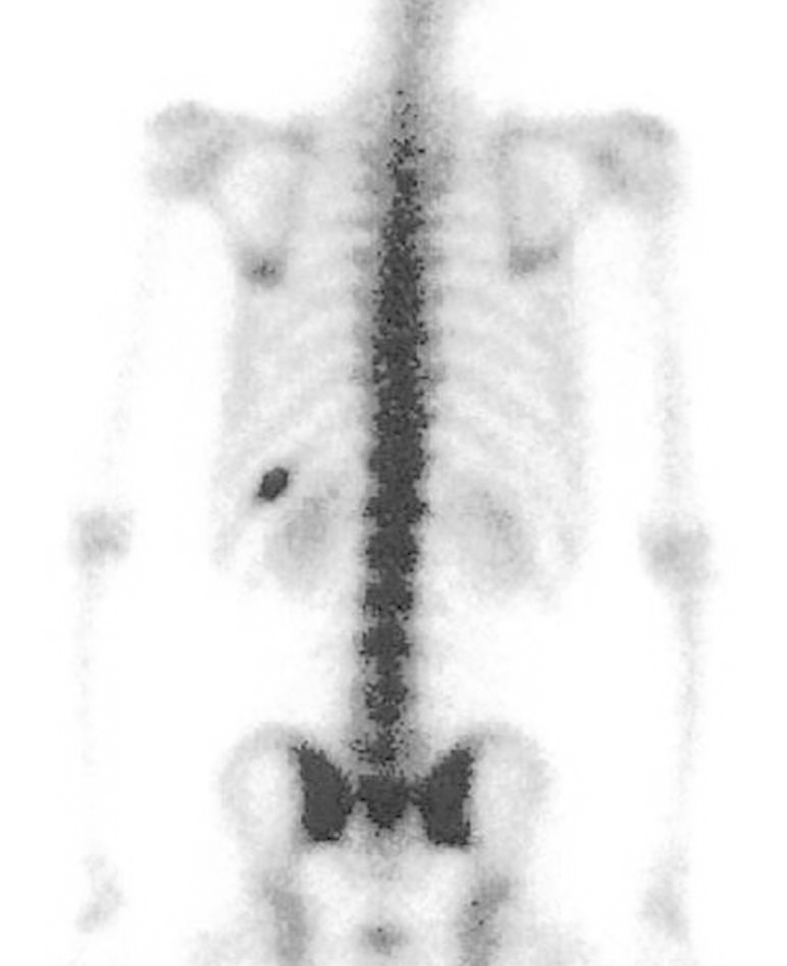
Bone scintigraphy revealing intense uptake at the site of the lesion.

**Figure 4:**
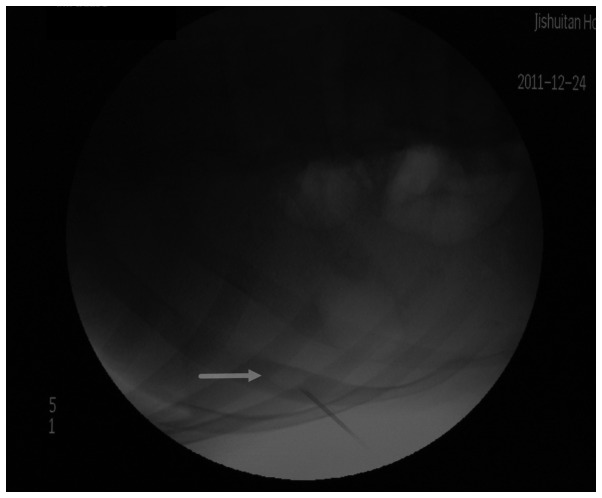
Lesion was located during surgery using a C-arm fluoroscopic device.

**Figure 5:**
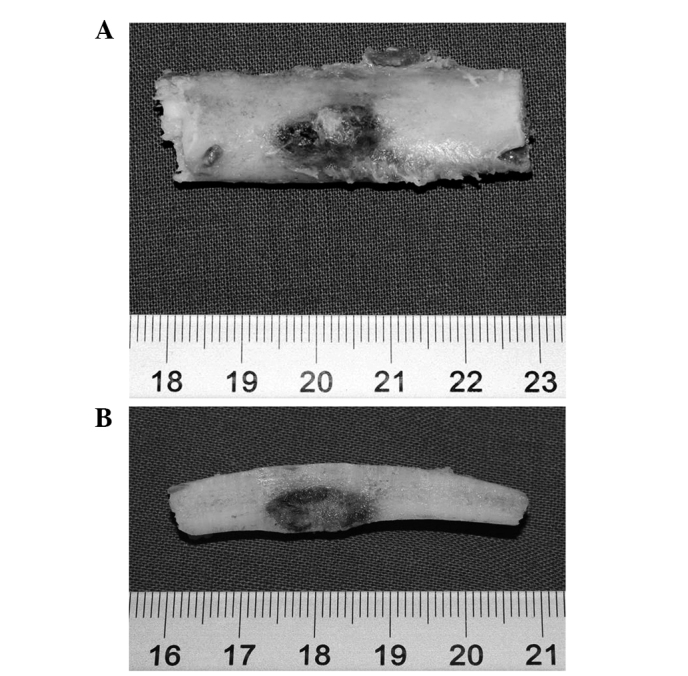
(A) Specimen with the lesion at the visceral side. (B) The cross-section view revealing the location of the lesion in the cortex.

**Figure 6:**
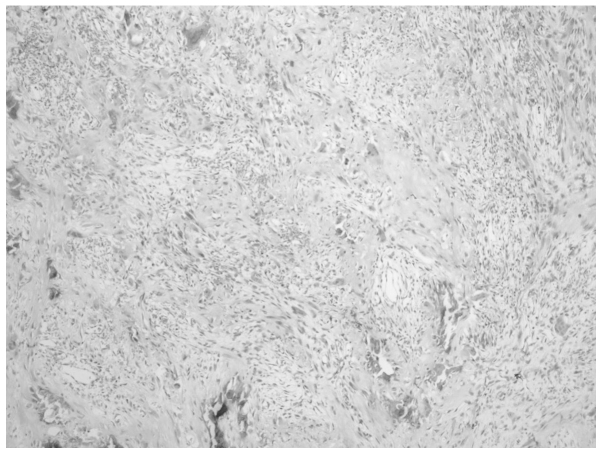
Photomicrograph revealing osteoid tissue in a background stroma of fibrovascular tissue (stain, hematoxylin and eosin; magnification, ×100).

A 22 year-old male presented with back pain in the right side that had been apparent for one year. The pain was intermittent and more severe at night, while it was reduced by taking aspirin. A local hospital was unable to establish a diagnosis and, thus, the patient was referred to the Beijing Jishuitan Hospital (Beijing, China) in December 2011. A fixed tenderness point was identified on the right side of the back. An X-ray image revealed a radiolucent lesion located in the tenth rib of the right side, which exhibited prominent sclerosis surrounding a central radiolucent nidus (Fig. 1). A computed tomography (CT) scan revealed clear central calcification of the lesion, which was located in the visceral side of the rib at the reconstructed view (Fig. 2). Technetium-99m medronic acid bone scintigraphy revealed a single focus of intense uptake in the posterior shaft of the right tenth rib (Fig. 3). A diagnosis of osteoid osteoma was subsequently established. A C-arm fluoroscopic device (BV Libra, Philips, Amsterdam, Netherlands) was used to locate and completely resect the lesion during surgery (Fig. 4). The specimen revealed that the visceral side of the rib was involved. In the cross section, the tumor was located in the cortex of the rib. The nidus and central calcification were clearly observed in the gross specimen (Fig. 5). A high-power photomicrograph (BX41, Olympus Corporation, Tokyo, Japan) revealed the presence of osteoid tissue in a background stroma of fibrovascular tissue and thin trabeculae inter-anastomosing with a single layer of osteoblasts. The intertrabecular space was filled with fibrovascular stroma (Fig. 6; stain, hematoxylin and eosin). The diagnosis was confirmed by the histological analysis. Following surgery, the chest pain was alleviated. At the time of writing, the patient remained tumor-free.

### Case 2

**Figure 7:**
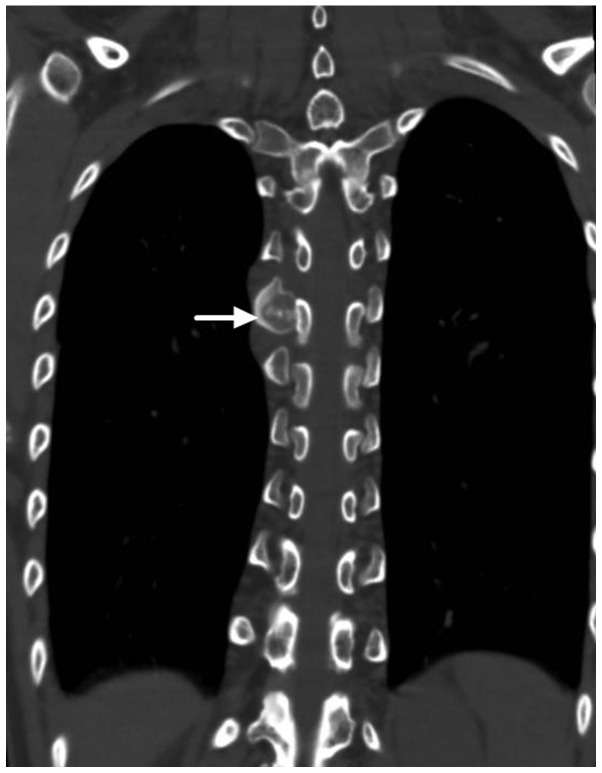

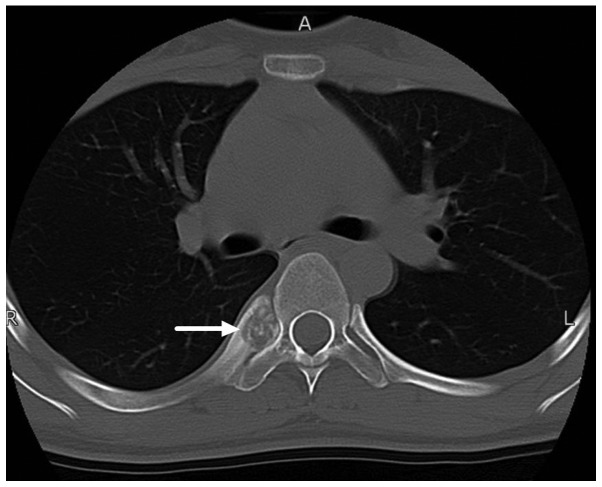
Computed tomography revealing the location of the lesion at the top of sixth right rib.

**Figure 8:**
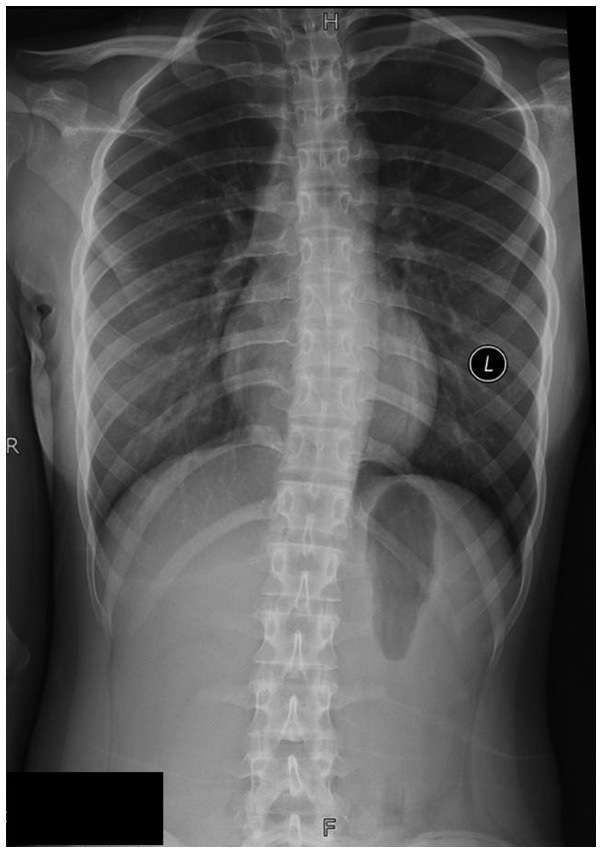
X ray revealing the presence of scoliosis in the spine plain film.

**Figure 9:**
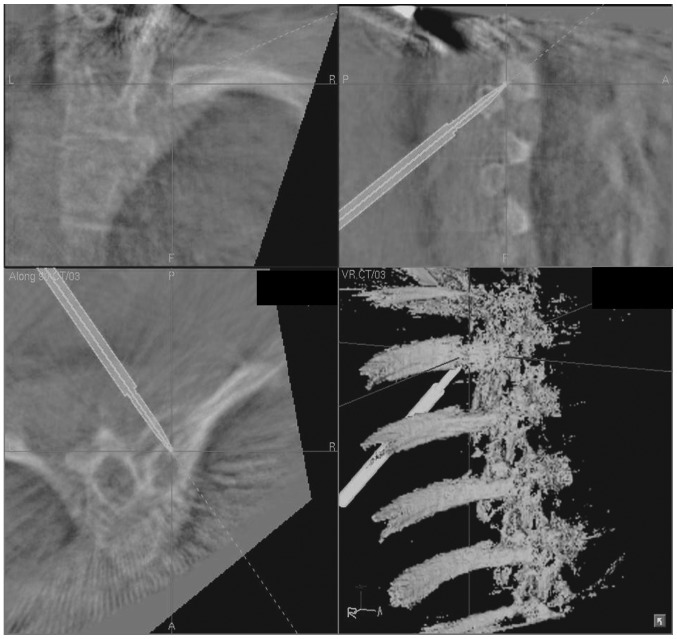
Intra-operative three dimensional C-arm-based navigation system was used to locate the tumor.

**Figure 10:**
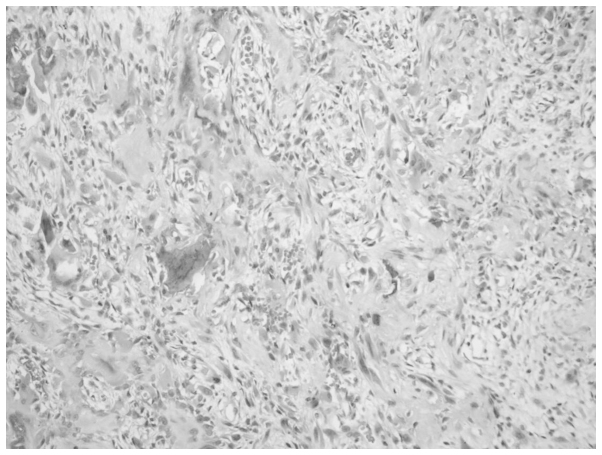
Photomicrograph revealing interconnecting trabeculae of woven bone lined prominently by osteoblasts (stain, hematoxylin and eosin; magnification, ×200).

In February 2011, a 16 year-old female was admitted to the Beijing Jishuitan Hospital. The patient presented with back pain that had been apparent for six months. A slight curve towards the left side of the patient’s back was observed. The patient complained of pain at night that was reduced by taking aspirin. A fixed tenderness point was identified in close proximity to the spine. A CT scan revealed a lesion that was located at the top of the sixth right rib with calcification present in the center (Fig. 7), while an X-ray scan revealed scoliosis of the thoracic spine (Fig. 8). An intra-operative three dimensional C-arm-based navigation system (REF 7700-500-000, Stryker, Kalamazoo, MI, USA) was used to locate and completely resect the tumor and curette the nidus (Fig. 9). The diagnosis was confirmed by histological analysis, which revealed interconnected, ossified bone trabeculae with abundant osteoblasts (Fig. 10; stain, hematoxylin and eosin, Tianhe Lien Company, Beijing, China). At the time of writing, the patient remained tumor-free.

## Discussion

Tumors of the rib are uncommon, constituting only 5–10% of all bone neoplasms. Osteoid osteomas are benign osteoblastic neoplasms, which are characterized by a well-demarcated core with a typical size of <1 cm and by a distinctive surrounding zone of reactive bone formation (2). An osteoid osteoma may occur anywhere in the cortex or medulla of the skeleton. However, the lesions usually affect the lower extremities. Although pain is the primary symptom of initial and recurrent disease (5), cases of osteoid osteoma without presence of pain have also been reported (6).

The most common types of tumors that affect the ribs are metastases and myelomas. Primary tumors of the ribs are uncommon. Therefore, the location of a tumor within the rib may help establish a differential diagnosis (7). Cartilaginous tumors frequently occur close to the costochondral junction, while rib sarcomas are more likely to present with symptoms of pain (8).

In total, <1% of osteoid osteomas affect the ribs (2). The defining symptom of osteoid osteoma is pain during the night that responds to nonsteroidal anti-inflammatory drugs and salicylates. The lesions most commonly involve the posterior or posterolateral shaft of the rib and, upon imaging, present with similar features to tumors located elsewhere in the body. Imaging typically reveals a small radiolucent lesion with a thick sclerotic margin of reactive bone. Due its accuracy in detecting the nidus, CT is the preferred imaging technique used for the assessment of osteoid osteomas (9–11). When the osteoid osteoma occurs in the posterior portion of the rib, it may lead to scoliosis (7), as observed in case 2 of the present study. In case 1, the tumor was located at the shaft of the rib, away from the spine; therefore, scoliosis did not occur. Small rib lesions may not be detected in the lung window of a chest X ray image, whereas CT scanning provides an improved field of view.

The differential diagnosis of painful lesions located close to the ribs should consider tumors of the bone. Aneurysmal bone cysts of the posterior vertebral elements, eosinophilic granulomas and osteoid osteomas are the most commonly occurring lesions (12). Due to the characteristic symptoms and the presence of the nidus in the CT scan, a diagnosis of other potential painful rib tumors was ruled out in the present study.

Complete surgical excision is the standard treatment method for osteoid osteoma and is usually offered to patients experiencing chronic and substantial pain that is not relieved by conservative treatment. In circumstances where excision is challenging, curettage is a good option. Removal of the nidus is the main aim of the treatment (13). Recent techniques for treating cases of osteoid osteoma involve the removal of the tumor by radiofrequency (14). However, as the lesions in the present study were located close to the pleura, the use of radiofrequency was deemed unsafe. Therefore, surgical excision and curettage were the selected treatment methods for cases 1 and 2, respectively. The diagnoses were confirmed by histological analysis.

In conclusion, osteoid osteoma of the rib is an extremely rare condition. The typical clinical symptoms and characteristic imaging features often allow for a clear diagnosis. The method used for the treatment of osteoid osteoma depends on the tumor location.
